# Endoplasmic Reticulum Stress-Regulated Chaperones as a Serum Biomarker Panel for Parkinson’s Disease

**DOI:** 10.1007/s12035-022-03139-0

**Published:** 2022-12-07

**Authors:** Katarzyna Mnich, Shirin Moghaddam, Patrick Browne, Timothy Counihan, Stephen P. Fitzgerald, Kenneth Martin, Ciaran Richardson, Afshin Samali, Adrienne M. Gorman

**Affiliations:** 1 Apoptosis Research Centre, University of Galway, Galway, Ireland; 2 School of Biological and Chemical Sciences, University of Galway, Galway, Ireland; 3 Department of Mathematics and Statistics (MACSI), University of Limerick, Limerick, Ireland; 4 Department of Neurology, University College Hospital, Galway, Ireland; 5 School of Nursing and Midwifery, University of Galway, Galway, Ireland; 6 School of Medicine, University of Galway, Galway, Ireland; 7 Randox Laboratories Ltd., 55 Diamond Road, Crumlin, Co. Antrim, Northern Ireland, BT29 4QY Ireland; 8 Randox Teoranta, Meenmore, Dungloe, Co. Donegal, Ireland

**Keywords:** Biomarker, Chaperone, Endoplasmic reticulum (ER) stress, Parkinson’s disease (PD), Serum

## Abstract

Examination of post-mortem brain tissues has previously revealed a strong association between Parkinson’s disease (PD) pathophysiology and endoplasmic reticulum (ER) stress. Evidence in the literature regarding the circulation of ER stress-regulated factors released from neurons provides a rationale for investigating ER stress biomarkers in the blood to aid diagnosis of PD. The levels of ER stress-regulated proteins in serum collected from 29 PD patients and 24 non-PD controls were measured using enzyme-linked immunosorbent assays. A panel of four biomarkers, protein disulfide-isomerase A1, protein disulfide-isomerase A3, mesencephalic astrocyte-derived neurotrophic factor, and clusterin, together with age and gender had higher ability (area under the curve 0.64, sensitivity 66%, specificity 57%) and net benefit to discriminate PD patients from the non-PD group compared with other analyzed models. Addition of oligomeric and total α-synuclein to the model did not improve the diagnostic power of the biomarker panel. We provide evidence that ER stress-regulated proteins merit further investigation for their potential as diagnostic biomarkers of PD.

**Graphical Abstract:**

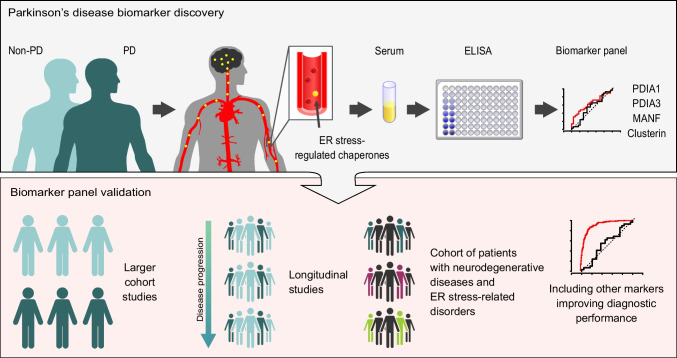

## Introduction

Parkinson’s disease (PD) is the second most common neurodegenerative disorder of the central nervous system. It is characterized by a progressive loss of dopaminergic neurons in the substantia nigra pars compacta and the accumulation of misfolded α-synuclein fibrils in glial and neuronal inclusions called Lewy bodies [1]. Making an accurate diagnosis of PD is difficult, time-consuming and is based on the clinical assessment of symptoms, medical history, physical and neurological examination, and responsiveness to the drug Levodopa. Imaging methods, such as magnetic resonance imaging (MRI), photon emission tomography (PET), and single-photon emission computed tomography (SPECT) are used to exclude conditions not associated with dopamine deficiency [1, 2]. Unfortunately, these methods are costly and some involve the exposure of patients to radiation. Thus, there is an urgent need for a non-invasive and rapid diagnostic test for PD [3]. Blood-based biomarker(s) monitoring pathological processes associated with the disease are economic, minimally invasive, and easily accessible methods that are readily used by clinicians for the diagnosis of various other diseases.

Neuropathological examination of post-mortem brain tissues from PD patients has revealed a strong association between PD pathophysiology and endoplasmic reticulum (ER) stress [4–9]. Studies using animal and cellular models of PD, including patient-derived induced pluripotent stem cells, indicate that ER dysfunction is an early event in PD pathogenesis contributing to neurodegeneration [6, 10, 11]. The ER plays a central role in the secretory pathway and it houses several resident chaperone proteins, which mediate the folding of transmembrane and secreted proteins [12]. These proteins contain Lys-Asp-Glu-Leu (KDEL)-like ER retention sequences and, while they are normally localized to the ER, they can be released from cells upon ER stress [13–16]. ER stress is induced by the accumulation of misfolded and unfolded proteins in the ER and it triggers the unfolded protein response (UPR). The UPR leads to the upregulation of ER-resident chaperone proteins, which restore proteostasis by facilitating proper folding and post-translational modifications of transmembrane and secretory proteins [17].

Systemic communication of stress signals between neurons and distal tissues has been recognized to affect non-cell autonomous control of homeostasis [18–20]. Indeed, stress proteins from neurons have been detected in the bloodstream [21, 22]. This offers an opportunity to uncover blood-based biomarkers of neurodegeneration that are related to ER stress. We hypothesized that ER stress-regulated chaperones that have been previously reported to (1) be upregulated in brain tissues of PD patients, (2) exhibit altered secretion from cells upon ER stress, and (3) have been detected in blood/cerebrospinal fluid (CSF) may have potential as blood-based biomarkers for PD. In this pilot study, we assessed levels of several ER stress-regulated chaperones in serum from PD patients and non-PD controls. We show that, while none of the analyzed proteins could independently discriminate between PD and non-PD groups, multiple logistic regression analysis and generation of a mathematical model comprising PDIA1, PDIA3, MANF, clusterin, and two confounding factors, age and gender, allowed for discrimination between the two diagnostic groups. These findings suggest that this panel of ER stress-regulated chaperones may have the potential as a diagnostic tool for PD, albeit with moderate sensitivity and specificity.

## Results

### Comparison of Serum Levels of Candidate Biomarker Proteins Between Non-PD and PD Groups

Based on our review of the literature to find proteins which met the selection criteria, chaperones PDIA1, PDIA3, MANF, GRP78, calreticulin, and clusterin were selected for validation as potential PD serum-based biomarkers. All of these proteins have been reported to be upregulated in PD [7, 9, 11, 23–28]. With the exception of clusterin, they are all KDEL-containing proteins that are residents in the ER [29, 30]. Clusterin is normally secreted through the ER-Golgi secretory pathway, but under conditions of ER stress it is redirected to the cytosol where it may be involved in the trafficking of misfolded proteins for degradation by the proteasome and/or autophagy [31, 32]. All of these proteins have been found in blood/CSF, which demonstrates their potential to be detected in serum [33–36].

**Fig. 1:**
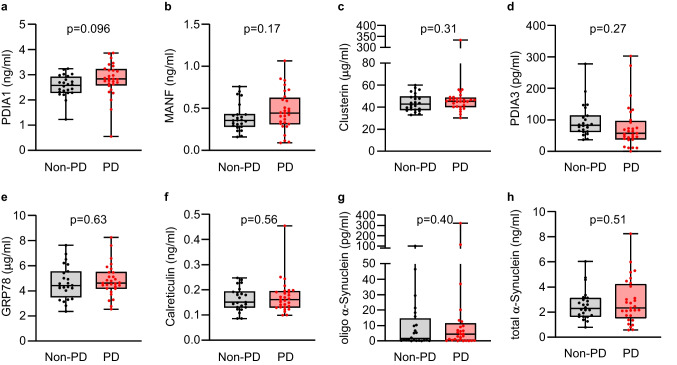
Serum concentrations of **a** PDIA1, **b** MANF, **c** clusterin, **d** PDIA3, **e** GRP78, **f** calreticulin, **g** oligomeric α-synuclein, and **h** total α-synuclein plotted as individual values in indicated diagnostic groups. The line through the middle of the boxes in box-and-whiskers plots corresponds to the median and the lower and the upper lines to the 25^th^ and 75^th^ percentiles, respectively. The whiskers extend from the minimum value at the bottom to the maximum value at the top

We performed enzyme-linked immunosorbent assay (ELISA) analysis to determine the concentrations of these proteins in serum taken from PD patients and non-PD controls. This revealed higher levels of PDIA1 (*p* = 0.096) (Fig. 1a), MANF (*p* = 0.17) (Fig. 1b), and clusterin (*p* = 0.31) (Fig. 1c), and reduced levels of PDIA3 (*p* = 0.27) (Fig. 1d) in PD patients, while GRP78 (*p* = 0.63) (Fig. 1e) and calreticulin (*p* = 0.56) (Fig. 1f) levels were similar in both groups. We noted that one of the PD values for clusterin (333.71 µg/ml) deviated very far from the mean value, and that if this sample was omitted from the calculation the mean changed from 54.69 to 42.56 µg/ml, which was similar to the mean for non-PD group (Table 1). We also examined levels of the oligomeric form of α-synuclein and total α-synuclein, since both have been previously flagged as promising biomarkers of PD. Levels of oligomeric α-synuclein were higher in serum from PD patients (*p* = 0.40) (Fig. 1g), while total α-synuclein levels were not different between diagnostic groups (*p* = 0.51) (Fig. 1h). However, the levels of examined proteins considerably overlapped between diagnostic groups, and the observed differences in mean protein levels between diagnostic groups did not reach the statistical significance, indicating that none of the tested proteins were able to distinguish between PD and non-PD groups as single biomarkers (Table 1).

**Table 1:** Serum concentrations of proteins in indicated diagnostic groups

	Non-PD (*n* = 24)	PD (*n* = 29)	*P*-value
PDIA1 (ng/ml), mean ± SD, (95% CI)	2.55 ± 0.44, (2.37–2.74)	2.81 ± 0.67, (2.56–3.07)	0.096
MANF (ng/ml), mean ± SD, (95% CI)	0.39 ± 0.16, (0.32–0.46)	0.47 ± 0.24, (0.34–0.56)	0.17
Clusterin (μg/ml), mean ± SD, (95% CI)	44.12 ± 7.82, (40.82–47.42)	54.69 ± 54.00, (34.14–75.23)	0.31
PDIA3 (pg/ml), mean ± SD, (95% CI)	*97.61 ± 54.43, (74.08–121.20)	78.12 ± 71.23, (51.03–105.20)	0.27
GRP78 (μg/ml), mean ± SD, (95% CI)	4.65 ± 1.34, (4.09–5.21)	4.82 ± 1.28, (4.34–5.31]	0.63
Calreticulin (ng/ml), mean ± SD, (95% CI)	*0.163 ± 0.047, (0.143–0.183)	0.172 ± 0.067, (0.147–0.198)	0.56
Oligo α-synuclein (pg/ml), mean ± SD, (95% CI)	11.49 ± 22.03, (2.18–20.79)	22.01 ± 61.97, (− 1.56–45.58)	0.40
α-Synuclein (ng/ml), mean ± SD, (95% CI)	2.57 ± 1.21, (2.06–3.07)	2.84 ± 1.80, (2.16–3.53)	0.51

Mean biomarker concentrations, standard deviations (SD), and 95% confidence intervals (CI) are given in serum of non-Parkinson’s disease (PD) and PD diagnostic groups. *23 non-PD samples

### A Panel of Biomarkers Discriminates PD Patients from Control Group

**Fig. 2:**
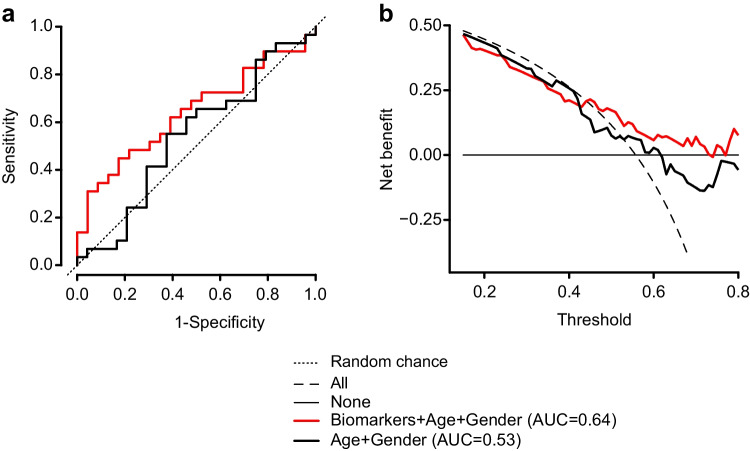
Diagnosis of Parkinson’s disease using serum PDIA1, MANF, clusterin, and PDIA3. **a** Receiver operating characteristic (ROC) curve and **b** decision curve analysis of PD versus non-PD groups

Levels of single biomarkers often show considerable overlap between diagnostic groups [37, 38]. In such circumstances, a combination of multiple biomarkers is a better strategy for obtaining a more accurate diagnosis [39, 40]. We performed multiple logistic regression analysis and identified a panel of four proteins PDIA1, PDIA3, MANF, and clusterin, which together with two confounders age and gender, provided the greatest discrimination of PD patients from the non-PD control group, with an accuracy measured by an area under the curve (AUC) of 0.64, calculated from the receiver operating characteristic (ROC) curve (sensitivity 66%, specificity 57%) (Fig. 2a). Decision curve analysis of this model including significant variables and the two confounders consistently demonstrated higher net benefit value in disease detection compared to “diagnose all,” “diagnose none,” and “diagnose based on age and gender” models (Fig. 2b).

**Fig. 3:**
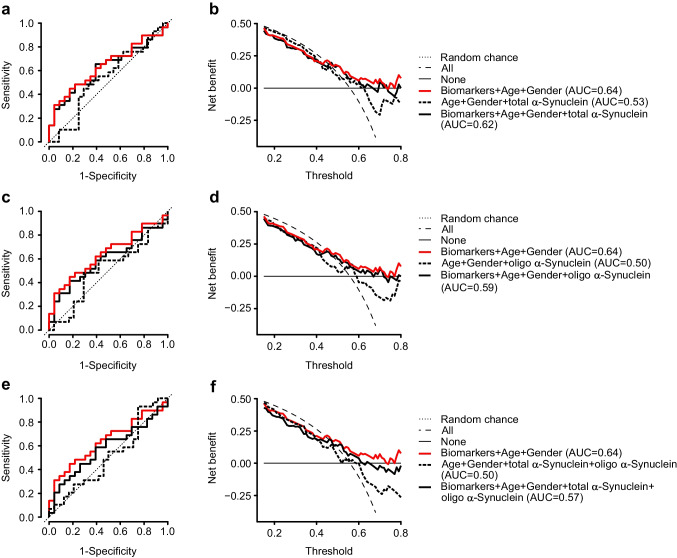
Lack of effect of total α-synuclein and oligomeric α-synuclein addition into biomarker panel on **a**, **c**, **e** ROC curve and **b**, **d**, **f** decision curve analysis of PD versus non-PD groups

Conversion of monomeric α-synuclein into oligomers and fibrils is a major neuropathological hallmark of PD and oligomeric α-synuclein has gained attention as a promising diagnostic biomarker [40–42]. We measured levels of oligomeric and total α-synuclein in serum (Table 1) and performed logistic regression analysis to test if α-synuclein can contribute to the performance of our biomarker panel. A combined assessment revealed that neither total α-synuclein (Fig. 3a, b), oligomeric α-synuclein (Fig. 3c, d) nor both combined together with the biomarker panel (Fig. 3e, f) improved the diagnostic discriminatory power and benefit of this biomarker panel.

## Discussion

This exploratory study is the first to report on the performance of a panel of ER stress-regulated chaperone proteins, analyzed in serum, in discriminating between PD patients and non-PD controls. We tested ER stress-related proteins that met our criteria of being increased in PD, displaying altered secretion upon ER stress and reported to be found in blood/CSF. We were able to detect each of the six proteins in serum from PD patients and non-PD controls, although none of the proteins tested were able to distinguish between PD and non-PD groups when used individually. However, multiple logistic regression analysis identified a panel containing PDIA1, PDIA3, MANF, and clusterin that could discriminate between PD and non-PD groups, with PDIA1 and MANF contributing most to the discriminatory power. This study underscores the value of combining multiple biomarkers and the potential of ER stress-regulated proteins as serum-based biomarkers for PD.

A recent systematic review provided evidence that biomarker panels increase diagnostic accuracy and outperform single biomarkers in the detection of Alzheimer’s disease [39]. Similar findings were described in a review focused on biomarkers for PD [2]. For example, a combination of oligomeric/total α-synuclein, phosphorylated α-synuclein, and phosphorylated tau in CSF discriminates PD from healthy controls significantly better than each biomarker individually [40]. Similarly, CSF neurofilament contributes to a panel of CSF α‐synuclein species in distinguishing PD from non-PD groups, even though neurofilament levels largely overlapped between the two groups [37]. In agreement with these studies, we found that a panel of ER stress-regulated biomarkers could discriminate PD and non-PD groups, while each protein individually could not.

Our findings show for the first time that ER stress-regulated proteins are worthy of investigation as serum biomarkers for PD. Indeed, higher levels of MANF and clusterin have already been reported in serum samples of PD patients [35, 43]. Increased concentrations of clusterin in CSF were reported to be predictive of PD [27]. Clusterin is an extracellular chaperone secreted through the ER-Golgi secretory pathway but its localization can be altered upon ER stress, with clusterin trafficking to the cytosol [31]. ERdj3 is another ER-resident protein whose secretion is elevated upon ER stress [44]. Unfortunately, we were unable to include ERdj3 in our analysis due to a lack of suitable ELISA. However, it would be very interesting to include this in the model.

Proteins, such as MANF, PDIA1, PDIA3, GRP78, and calreticulin are normally ER resident chaperones due to the presence of ER-localizing C-terminal KDEL-like sequence [30]. KDEL proteins are recognized by KDEL receptors in the Golgi and recycled back to the ER [45]. Upon ER stress, the increased expression of KDEL-containing ER resident proteins can overwhelm the KDEL-dependent retrieval system which results in their secretion from cells [30]. Moreover, imperfect KDEL sequences, such as the C-terminal RTDL sequence in MANF, can lead to poorer ER retention and easier diversion of the protein. KDEL-like sequences have been found in more than 70 human proteins [29, 30]. Given that biomarker panels exhibit improved performance over single biomarkers, it is worth considering whether other KDEL proteins could contribute to the enhanced performance of the panel.

The diagnostic accuracy of the ER stress-regulated biomarker panel was moderate at best, with an AUC of 0.64. This indicates a need for further panel evaluation to better understand the dynamics and confounders that affect its performance. For example, age-related changes in chaperone expression might occur due to a decline in proteostasis in neurons [46]. Biological sex is also an important factor contributing to the clinical features of PD [47]. Indeed, we observed that age and gender contributed to the performance of the biomarker panel and therefore, they were included in the analysis. By incorporating sex into the model, the unbalanced male/female ratio in the samples collected was also taken into account. It is possible that other factors, for example, disease duration and severity, may influence chaperone levels in the serum of PD patients. For example, it has been reported that MANF levels in serum are higher in PD patients classified as depressed based on Beck Depression Inventory scoring [35]. A better understanding of confounding factors could help to explain why some patients had significantly higher values for specific biomarkers, like clusterin, than the group mean values. This needs to be investigated further in a larger cohort study.

Furthermore, combining biomarkers reflecting the multiple neuropathological processes that are associated with PD may improve the diagnostic accuracy of the biomarker panel. Apart from ER stress, inflammation, lysosomal dysfunction, metabolic impairment, aberrant autophagy, amyloid pathology, tauopathy, and synucleinopathy have also been implicated in PD [2]. Interestingly, ER stress impacts most of those processes [48, 49]. For example, ER stress has been reported to induce α-synuclein oligomerization [50, 51]. Previously, it has been suggested that oligomeric α-synuclein in serum might be a potential biomarker for the diagnosis of PD [41, 52]. The reports on total α-synuclein levels in the blood of PD patients and non-PD controls are conflicting [53–55]. We did not observe significant differences in levels of total and oligomeric forms of α-synuclein between diagnostic groups. The inclusion of total and oligomeric α-synuclein in our model did not improve the performance of the biomarker panel in discriminating between PD and non-PD groups.

In conclusion, the data presented here show for the first time a panel of PDIA1, PDIA2, MANF, and clusterin that could be useful in the diagnosis of PD. This pilot study provides a rationale for further validation of ER stress-regulated chaperone proteins in a larger cohort study as well as longitudinal studies to assess the dynamics of changes as a function of PD stage and duration. It will be important to evaluate the model in cohorts of patients representing a spectrum of neurodegenerative diseases and ER stress-related disorders. The results also underscore the potential added benefit of including other biomarkers in the panel in order to improve its diagnostic performance.

## Methods

### Participant Recruitment and Assessment

This cross-sectional pilot study was approved by the University Hospital Galway Research Ethics Committee and written informed consent was obtained from the subjects. Blood samples were collected from 29 idiopathic PD patients and 24 non-PD controls. Patients diagnosed with PD before the onset of the study were recruited from a tertiary referral movement disorders clinic and all patients were diagnosed with idiopathic Parkinson’s disease by a movement disorder specialist in accordance with current Movement Disorder Society (MDS) clinical diagnostic criteria [56]. Hoehn & Yahr (H&Y) staging and Mini-Mental State Examination (MMSE) score assessments were completed for the enrolled patient on the day of blood collection. Patients were eligible for inclusion if they were diagnosed with idiopathic PD by a movement disorders specialist, with no signs of dementia (MMSE score ≥ 24), and moderate disease severity (H&Y stages 2 and 3). Clinical data, such as disease duration and medication, were obtained. Unrelated non-PD subjects or spouses of patients were enrolled from the local community. Volunteers with neurological disorders on cytotoxic drugs or a family history of neurodegenerative diseases were excluded. The demographic and clinical characteristics of the enrolled subjects listed in Table 2 show the distribution of age and sex between the diagnostic groups.

**Table 2:** The demographic and clinical characteristics of the enrolled subjects

	PD (*n* = 29)	Non-PD (*n* = 24)	*P*-value
Age (years), mean ± SD	68.5 ± 9.94	64.3 ± 10.97	0.15
Gender (F/M)	13/16	15/9	0.21
MMSE score, mean ± SD	28.73 ± 1.55	nd	nd
H&Y stage (number per stage 2/3)	12/17	n/a	nd
PD duration (months), mean ± SD	124.4 ± 60.09	n/a	nd

Demographic data are given as mean ± SD except dichotomous values. *F*, female; *H&Y*, Hoehn and Yahr scale; *M*, male; *MMSE*, mini-mental state examination; *n/a*; not applicable; *nd*, not determined

### Collection and Processing of Human Serum Samples

Venous blood was collected by a trained phlebotomist into BD Vacutainer serum tubes (Becton, Dickinson, NJ, USA; #367,895) containing silica for clot activation. Samples were left to coagulate in a dark at room temperature for 1 h. Tubes were centrifuged at 1300 × g for 10 min. Serum was collected, aliquoted into polypropylene 0.5 ml tubes, and stored at − 80 °C within 50 min from centrifugation. Serum samples were validated for hemolysis.

### The Biomarker Selection Process

PubMed was used to search English language publications. The initial database search used the following search terms: (endoplasmic reticulum stress OR the unfolded protein response) AND (protein secretion OR secretory proteins). The literature search identified two sets of proteins whose secretion is under the control of ER stress: (1) proteins that are normally resident in the ER, due to the presence of a C-terminus KDEL-like retention sequence but have been demonstrated to be released from cells upon ER stress; and (2) secretory proteins that are folded in the ER and whose secretion is altered upon ER stress. We searched through the list of proteins to identify chaperones. From the list of proteins with KDEL-like motif [29, 30], we selected chaperones PDIA1, PDIA3, GRP78, calreticulin, MANF, and CDNF, because they were previously reported to (1) be upregulated in brain tissues of PD patients [7, 9, 11, 23–28], (2) exhibit altered secretion from cells upon ER stress [13, 57–59], and (3) have been detected in blood/CSF [33–36, 60, 61]. From the list of secreted chaperones, only the localization of clusterin and ERdj3 have been reported to be altered upon ER stress [32, 44]. We tested clusterin, for which higher levels have been reported in serum and CSF samples of PD patients [27, 43]. We did not analyze CDNF or ERdj3 due to the lack of a suitable ELISA that would enable us to measure protein levels in serum without a matrix effect.

### Enzyme‐Linked Immunosorbent Assay (ELISA)

Protein concentrations in serum were measured by enzyme‐linked immunosorbent assays: PDIA1 (Abbexa, Cambridge, UK; abx152685), MANF (Abcam, Cambridge, UK; ab215417), PDIA3 (Abbexa; abx252930), GRP78 (Enzo, NY, USA; ADI-900–214), Calreticulin (Abbexa; abx250954), Clusterin (BioVendor, Heidelberg, Germany; RD194034200R), α-synuclein (Invitrogen, CA, USA; KHB0061), and oligomeric α-synuclein (Analytic-Jena, Jena, Germany; BM-847–0,104,000,108) according to the manufacturer’s protocols. Protein standard dilutions and blank (1 × dilution buffer) were prepared and 100 μl of each were added in duplicate to a pre-coated plate. The serum samples were either undiluted (for detection of PDIA1, PDIA3, calreticulin, oligomeric α-synuclein) or diluted in a sample diluent buffer (1:25 for GRP78, 1:8 for MANF, 1:2 for α-synuclein, 1:3,000 for clusterin) and 100 μl were added in duplicate into wells. For the GRP78 ELISA, 50 μl of antibody were added into each well, except the blank wells. The plates were incubated at room temperature for 1 h with shaking. The wells were washed 5 times with 300 μl 1 × wash solution except for the GRP78 ELISA. After the final wash, any remaining solution was removed. A secondary antibody solution was added into each well (100 μl for detection of MANF, clusterin, calreticulin, PDIA1, and PDIA3 or 50 μl for detection of GRP78). The plates were incubated for 1 h at room temperature on a shaker. The wells were washed 5 times. Substrate solution (100 μl or 200 μl for GRP78 detection) was added and plates were incubated for up to 30 min at room temperature with shaking. The plates were sealed at every incubation step. The stop solution (100 μl or 50 μl for GRP78 detection) was pipetted into each well. The absorbance at 450 nm was measured using a VICTOR3™ Multilabel Plate Reader (Perkin Elmer, MA, USA). For the calculation of protein concentration, the mean absorbance of the blank was subtracted from all readings. The protein concentrations were interpolated from the standard curve and multiplied by the respective dilution factor. The standard curve was generated by plotting the mean absorbance of the standards against the known concentration of the standards in a logarithmic scale, using the four-parameter algorithm.

### Statistical Analysis

Quantitative variables were compared using an unpaired two-tailed *t*-test with Welch’s correction. *P*-values were considered significant if *p* < 0.05. Patient information including serum levels of ER stress-regulated chaperones were used to build a statistical model to predict PD. Biomarker concentrations were adjusted between plates for the biomarkers that showed a significant difference between the plate means.

For oligomerized α-synuclein, 31% of the values were below the detection limit of the analytical procedure. These values called censored were different from missing values as they lie between zero and the detection limit. Cohen’s method was used for the imputation of data below the detection limit considering the sample size and the percentage of censoring [62]. The data were imputed using the inverse cumulative normal distribution with the adjusted sample mean and standard deviation using the maximum likelihood estimation.

The stepwise variable selection technique was used to identify a combination of biomarkers which could distinguish Parkinson’s disease patients from the control group [63]. The effects of selected biomarkers were modelled using logistic regression [64]. Ten-fold cross validation was used for internal validation of the models to confirm that no patient was used to both develop and test the model [65]. Ten-fold cross validation involves randomly dividing the data into ten evenly sized subgroups (fold). The data from the first nine folds were used for modelling and applied to the tenth fold as the validation set. The model building and validation process was repeated ten times with each fold of patients used once as the validation set. The accuracy of the models was determined using the area under the curve (AUC) calculated from the receiver operator characteristic (ROC) by plotting the sensitivity and specificity at each of its risk thresholds [66]. The closer the AUC value to 1, the better the panel of biomarkers can distinguish PD patients from the control group. Decision-curve analysis was also undertaken to examine the potential net benefit of the application of each model where a higher net benefit value shows improvement in disease detection [67].

## Data Availability

Anonymized data that support the findings of this study are available on request from the corresponding author for purposes of replicating procedures and results. The data are not publicly available due to containing information that could compromise research participant consent.
